# Epicardial adipose tissue measured by magnetic resonance imaging predicts abnormal adenosine stress cardiovascular magnetic resonance imaging and future adverse cardiovascular events

**DOI:** 10.1186/1532-429X-15-S1-P274

**Published:** 2013-01-30

**Authors:** Jason J Lamanna, Chesnal D Arepalli, Agathi R Vrettou, Emily L Ebert, Arash Harzand, Emir Veledar, John N Oshinski, Stamatios Lerakis

**Affiliations:** 1 Cardiology, Emory University, Atlanta, GA, USA; 2 Medicine, Emory University, Atlanta, GA, USA; 3 Radiology, Emory University, Atlanta, GA, USA; 4 Biomedical Engineering, Emory University/Georgia Institute of Technology, Atlanta, GA, USA

## Background

A growing body of evidence demonstrates a quantitative association between Epicardial Adipose Tissue (EAT), cardiometabolic risk factors and measures of coronary artery disease (CAD). It is still unclear, however, if EAT is predictive of abnormal functional stress tests and clinical outcomes. The aim of this study is to elucidate the relationship between the total volume of EAT, the detection of ischemia and/or infarct with Adenosine Stress Cardiovascular Magnetic Resonance imaging (AS-CMR), and combined future adverse cardiovascular events.

## Methods

Sixty-one patients, who presented to the emergency department with symptoms suggestive of CAD and were evaluated by AS-CMR, were enrolled in the study. Total volume of EAT was quantified with axial Half Fourier Acquisition Single shot Turbo spin Echo (HASTE) MR images using a rapid semi-automated threshold technique and commercial software. EAT volume was normalized to body Weight (EAT/W). The detection of ischemia and/or infarct by AS-CMR was considered an abnormal exam. Patients were followed by medical record review. The primary end-point was the composite of measured future adverse cardiovascular events: cardiac death, nonfatal myocardial infarction, obstructive CAD visualized with invasive coronary angiography, cerebrovascular accident, heart failure, arrhythmia, or recurrent chest pain of cardiac origin requiring hospital admission.

## Results

Total EAT volume and EAT/W were significantly increased in the 4/61 (6.6%) patients with abnormal AS-CMR compared to the patients with a normal exam (157 ± 42 mm3 vs. 97 ± 39 mm3, p = 0.0046 and 1.9 ± 0.6 vs. 1.2 ± 0.6, p = 0.0091, respectively). Total EAT volume and EAT/W were also significantly increased in patients reaching the primary endpoint (16/61, 26.2%) compared to those without observed events (129 ± 43 mm3 vs. 91 ± 37 mm3, p = 0.0014 and 1.6 ± 0.6 vs. 1.1 ± 0.5, p = 0.0040, respectively).

## Conclusions

Patients with an abnormal AS-CMR exam had significantly increased volume of EAT. Furthermore, patients with increased volume of EAT experienced significantly more future adverse cardiovascular events. Based on these results, EAT volume measured by CMR may become an important prognostic factor for cardiac risk stratification that should be measured and reported with every CMR study.

## Funding

N/A

